# Affective Neuronal Selection: The Nature of the Primordial Emotion Systems

**DOI:** 10.3389/fpsyg.2012.00589

**Published:** 2013-01-09

**Authors:** Judith A. Toronchuk, George F. R. Ellis

**Affiliations:** ^1^Department of Psychology, Trinity Western UniversityLangley, BC, Canada; ^2^Department of Biology, Trinity Western UniversityLangley, BC, Canada; ^3^Department of Mathematics, University of Cape TownCape Town, South Africa

**Keywords:** disgust, dominance, phylogenetic origins, primordial emotional systems, emotions, emotion and cognition

## Abstract

Based on studies in affective neuroscience and evolutionary psychiatry, a tentative new proposal is made here as to the nature and identification of primordial emotional systems. Our model stresses phylogenetic origins of emotional systems, which we believe is necessary for a full understanding of the functions of emotions and additionally suggests that emotional organizing systems play a role in sculpting the brain during ontogeny. Nascent emotional systems thus affect cognitive development. A second proposal concerns two additions to the affective systems identified by Panksepp. We suggest there is substantial evidence for a primary emotional organizing program dealing with power, rank, dominance, and subordination which instantiates competitive and territorial behavior and is an evolutionary contributor to self-esteem in humans. A program underlying disgust reactions which originally functioned in ancient vertebrates to protect against infection and toxins is also suggested.

## Introduction

Cognitive development of individuals, we have suggested in a previous paper, proceeds in part due to influences of primary emotional operating systems which act collectively as fitness criteria guiding further neuronal development (Ellis and Toronchuk, 2005). Specifically we suggested that Panksepp’s (1998, 2001) formulation of affective neuroscience can be seen as a complement to the theory of neural Darwinism as proposed by Edelman (1989, 1992). Important aspects of neural development could be explicated by linking the relevant concepts proposed by Edelman and Panksepp. Although we used the term Affective Neuronal Darwinism in previous papers, we will now adopt the term Affective Neuronal Selection in light of recent critique of Edelman’s theory as being selectionist, but not truly Darwinian (Fernando et al., 2012). Our hypothesis provides a possible extension of the role of Hebbian connectionism in guiding brain development, and it also integrates neuroanatomy with evolutionary psychiatry and ethology. Neural plasticity in this model would be guided by a process of selection of the most effective pathways based on the valences provided by primary emotional systems. Although the notion of valence (usually as approach/avoidance or pleasure/pain) is common in theories of emotion, we use the term as referencing reward and punishment, which most likely reflect internal states or markers (see discussions in Prinz, 2004, 2010; Rolls, 2005). In this way primordial emotions would provide at least some of the underlying processes for acquisition of cognitive processes as well as certain psychopathologies.

Our proposal contributes to the historical debate on the interdependence of cognition and emotion (Zajonc, 1980; Lazarus, 1984) by examining the role of primordial emotion-related processes in the development of cognition. Cognition and emotion are now recognized as exerting mutual influence with such entanglement that the divide between the two seems at times artificial (Ray and Zald, 2012). The role of non-conscious emotional processing and resulting biasing of cognitive processes has thus become a major focus, in addition to the more traditional concept of cognitive control of emotional regulation. The heuristic principles in affective science, therefore, involve ontogeny and phylogenetic continuity (Tamietto and de Gelder, 2010; de Gelder et al., 2011).

Neurotrophins, such as brain derived neurotrophic factor (BDNF), and monoamines such as serotonin, play critical roles in neural selection by promoting neural plasticity, activity dependent refinement of neural networks, and selection of simultaneously active neurons for survival (e.g., Lauder, 1993; Poo, 2001; Hua and Smith, 2004). Early activity in emotional circuits may thereby influence the wiring of later developing brain areas. Under adverse conditions, however, complex interactions between neurotrophins, monoamines, cytokines, and environmental influences may result in psychiatric or neurodegenerative disorders (reviewed in Castrén, 2005; Duman and Monteggia, 2006; Goodyer, 2008; Dinan, 2009). For example dopamine knockout mice have reduced BDNF in the frontal cortex which may lead to reduced plasticity (Fumagalli et al., 2003). Serotonin, perhaps the earliest transmitter present in embryos, plays several critical roles during neural development which leave permanent effects (reviewed in Whitiker-Azmitia, 2001). Because BDNF (Gordon et al., 2003), and nerve growth factor (Alfonso et al., 2004) can be modulated by activations of emotional systems, the wiring of brain circuitry may be widely influenced by activation in emotional circuits during early life. The immune system is also implicated in development, plasticity and behavioral disorders (e.g., Bauer et al., 2007; Müller and Schwarz, 2007; Bilboa et al., 2008; Baharnoori et al., 2009; Kemeny, 2009; McAfoose and Baune, 2009; Miller, 2009). Prenatal stress causing endocrine and immune changes in neurotransmitter systems can alter the developmental course of the brain increasing the risk of various disorders (e.g., Ansorge et al., 2007). Early childhood trauma has similar effects (Nemeroff et al., 2006; Goodyer, 2008). Both prenatal and postnatal activation of the monoamine, endocrine, and immune systems may thereby alter brain plasticity, and although this may occur initially at lower brainstem levels, ultimately the developmental course of cortical areas may be altered.

Thus primary emotional systems, influenced by endocrine and immune functions, mediate the core nature of the selection criteria guiding the refinement of synaptic connections to provide the emotional palette that guides individual brain development (Ellis and Toronchuk, 2005). This mechanism ultimately influences learning, cognitive, and social development. It follows that elucidation of the specific nature of these systems may shed light on the way the brain functions and structures itself.

Panksepp has described seven “primitive emotional operating systems that exist in limbic and reptilian areas of the brain” (Panksepp, 1998, p. 52). These hierarchically organized executive operating systems give rise to specific valenced affective states which guide flexible behavior while interacting with several layers of non-specific perceptual, attentional, and cognitive processes. Panksepp stresses that primary emotions, in contrast to secondary (discussed below), include instantiation in the phylogenetically ancient medial and ventral brainstem pathways rich in visceral innervation which utilize a variety of visceral neuropeptides (Panksepp, 1998, 2003a). While concurring with Panksepp, we propose that these primeval valenced systems should not only be capable of altering evolutionary survival rates of organisms, but be effective at the developmental level in determining which synapses survive in individual brains in accord with Edelman’s neural Darwinism. We propose an integrative perspective emphasizing both individual ontogeny *and* a broad evolutionary narrative for identification of primary systems with a resulting *proposal for a more complete set of primary emotional systems*.

Our list of primordial emotional operating systems takes into account previous proposals by authors from Darwin onward (e.g., Ekman, 1972; Izard, 1992; Damasio, 1999), but is based primarily on the comprehensive studies of Panksepp. Although Ekman (1992) and others also invoke evolution, most criteria for basic emotions have emphasized human facial or semantic features and have assumed primarily a communicative role for emotions (see Sabini and Silver, 2005, for a critique of Ekman’s criteria). Such approaches begin with human subjective experience and then search for *ad hoc* supporting data from other mammals rather than starting with a framework informed by vertebrate evolution. The type of phylogenetic approach to emotions described by Lawrence and Calder (2004) is necessary, but should extend beyond the mammalian order.

## Basic Insights from Evolutionary Psychiatry

Two key issues emerge from discussions in evolutionary psychiatry (Price, 1967; Stevens and Price, 2002; also, e.g., Gilbert and Allan, 1998; Price et al., 2007).

1. Evolutionary pressures led to the development of various psychological traits that are experienced by us as emotions. These give rise to behavior patterns which originally enhanced survival. For example the need for reproductive effectiveness results in emotional states of desire and bonding that promote propagation of genes. This is a clear statement of the causal efficacy of emotional systems in terms of affecting the evolutionary process.
2. *Many psychiatric disorders result from malfunctioning of these evolutionary adaptive mechanisms; hence the nature of such disorders is evidence of the nature of the underlying emotional mechanisms*. This means we can attempt to relate basic emotional systems to specific evolutionary adaptations using psychiatric data as supportive evidence.

Stevens and Price (2002) emphasize the pathologies that result from failures in the *attachment* and *rank systems*. These archetypal systems can “function healthily when evoked in appropriate circumstances, but either can give rise to pathology when their goals are frustrated or when they are inappropriately activated” (Stevens and Price, p. 50). The rank or power/dominance system “enables an individual to assess whether a rival is weaker or stronger and to produce the appropriate response” (p. 75). This system has been well-studied in fish, reptiles (MacLean, 1990), and even crayfish (Panksepp and Huber, 2002), attesting to its ancient lineage. In line with these concepts from evolutionary psychiatry, we develop below a systematic proposal which incorporates a *rank/power/dominance system*.

## The Underlying Propositions and Criteria

Our proposals are based on the following series of causal mechanisms:

1. Emotional systems emerged because they were causally effective in changing behavioral patterns.
2. Emotional systems were selected for in terms of their enhancement of survival resulting in the evolution of primary emotional systems which then come to shape the development of both intellectual capacities and secondary emotions.
3. Survival of individuals may be enhanced by group membership that allows benefits in terms of food procurement, protection, learning, and eventually culture.
4. To make group membership effective, there must be both group cohesion mechanisms, and mechanisms for resolving conflict and resource allocation tensions. These necessitate some form of communication between members.
5. The attachment systems and the power/dominance system evolved to meet the needs of individuals living in groups; they supplement the basic systems for survival and learning.
6. Humans experience, particularly through subjective feelings thereby induced, emotional operating systems whose mechanisms lie beneath psychological and developmental events without awareness of their evolutionary origins and function.

Following on this, one can propose a clear set of criteria characterizing primary emotional systems based on evolutionary aspects. We propose that a well-established primary system should have *all* the following characteristics:

C1. *Concept:* It corresponds to a specific range of human affects and characteristic behaviors, associated with clear eliciting stimuli and with universal affective outcomes, expressed in specific bodily behavior which may include facial expressions.
C2. *Structure:* It is effective through specific neural circuitry affected by a combination of transmitters, neuromodulators, hormones, and cytokines and will ultimately be traceable by neuroanatomical techniques. Each primary emotional circuit supervenes on a distinct *pattern* of neural pathways rather than on an exclusive set of structures. These circuits comprise distributed networks extending from brainstem to cortex; each is integrated with the pathways of other primary emotions which may utilize overlapping pathways.
C3. *Function*: Each primordial emotion system enlivens immediate affective functioning and because a unique combination of neurochemicals affects each core system, each can functionally take part in neural selection during ontogeny of the brain.
C4. *Development*: Development of emotional systems will be initiated by multiple genes and therefore susceptible to alteration by mutation or deletion. Environmental influences further affect expression of these genes.
C5. *Origin*: On a phylogenetic level, each system can be associated with adaptations expressed in cladistic homologous traits (Griffiths, 1997, p. 213), and hence can be clearly related to an evolutionary process.
C6. *Occurrence*: These primordial emotional systems, associated with homologous systems and evolutionary precursors, remain universally influential in humans; this enables a correspondence of the features listed above (2–5) between humans and other vertebrates.
C7. *Outcome*: Dysfunctional aspects can be associated with behavioral or psychiatric disorders, whose nature is related to deletion of functions and/or disinhibition of lower level components of its circuitry, or to over-activation of these functions; and hence such disorders shed light on normal function.

One key problem is to separate what Panksepp (2000, 2005) refers to as reflexive and sensory affects from true primary emotional operating systems. We describe these operating systems as action promoting valenced states with distinct circuitry and neurochemistry, the initiation of which can precede or anticipate potential environmental events and the consequences of which can outlast the precipitating conditions. In contrast reflexive affects (e.g., startle) are closely time-locked to triggering stimuli. An emotion, therefore, is a *superordinate* program which orchestrates and integrates the activities of various functional subprograms including reflexive affects, perception, cognitive appraisals, and feeling states (Cosmides and Tooby, 2000). Core emotional operating systems underlie complex, extremely flexible reactions by activating or inhibiting autonomic, hormonal, and/or somatic changes. The specific combination of behavioral components will depend on context, experience, and eliciting stimulus.

A second key problem is to differentiate primary from secondary emotional systems. In principle items C1, C2, C3, and C4 should be universally consistent in core systems, but not necessarily consistent for each secondary emotion. Secondary emotions arise from interactions between primary emotional circuitry and cognitions instantiated in neocortex (Panksepp, 2000; Prinz, 2004, p. 144–147) whereas the primordial emotions are largely dependent on subneocortical structures. Indeed the cortex is not always necessary for emotion based functions (de Gelder et al., 2011; Tamietto et al., 2012). We do not expect secondary emotions to occur with as great universal consistency of structure and function nor do we expect widespread occurrence in other mammals. Although there is no single criterion for discriminating primary systems from secondary emotions, our proposal is based on converging evidence from several methodologies (see Prinz, 2004, p. 90). We suggest that a good proposal can be made for a primary emotional system when all of Items C1–C3 and C5, C6 above occur; and is indisputable if the full set of items C1–C7 have been established, with the cladistic link C5 being especially important. Mere *existence* of a speculative evolutionary explanation is not sufficient, but does lend weight to the consideration that we are dealing with a primordial system.

## The Relation between Needs and Emotional Systems

In order to survive and pass on their genes all organisms require the fulfillment of certain basic conditions or needs. The development of emotional systems occurs in response to these biological, safety, and social needs thus promoting goal-seeking behavior. The primary emotions identified by Panksepp (1998) are^1^:

E1: The SEEKING system: incentive motivation, seeking, expectancy.
E2: The RAGE system: rage/anger.
E3: The FEAR system: fear/anxiety.
E4: The LUST systems: lust/sexuality.
E5: The CARE system: providing parental care/nurturance.
E6: The PANIC system: panic/separation, need of care.
E7: The PLAY system: rough-housing, play/joy.

Table 1 summarizes our proposed completion of Panksepp’s list, together with associated functions and relation to evolutionary needs. *Each basic developmental need has been matched during evolution by a corresponding core operating system that has become genetically programmed in* *accord with the above theses*. We further propose that *these primordial systems, adapted for survival needs of individual organisms, embody selection criteria underlying neural connections thereby determining brain development*. Thus they provide affective valence (as reward/punishment markers) influencing cognitive and social development (as discussed in Ellis and Toronchuk, 2005). The first group of systems relate primarily to the functioning of individuals, and the second to functioning of individuals in social groups. Although we have used this distinction as a broad classification, we stress that it is not essential to our proposal. (Compare, e.g., Buck’s (1999) grouping of selfish vs. social biological emotions corresponding to right vs. left hemisphere activation.)

**Table 1:** **Evolutionary needs, and the emotional systems that have evolved to meet them**.

Evolutionary needs met	Primary emotional system	Works with:	Functions
**INDIVIDUAL NEEDS**			
Basic Functioning	E1: SEEKING system	E2–9	Situation evaluation, incentive salience, hedonic appraisal, facilitates learning
Basic survival	E2: DISGUST system (repulsion, avoidance)		Avoiding harmful foods, substances, environments
	E3: RAGE system	E4,E9	Defense: protection of organism, resources, and conspecifics, limiting of restraint on movement
	E4: FEAR System	E3,E9	Defense: flight, limiting of tissue damage
**SOCIAL NEEDS**			
Reproduction	E5: LUST system (sexual desire, satiation)	E6,E7	Ensuring procreation, enhancement of bonding
Group cohesion: bonding and development	E6: PANIC/attachment (affiliation, separation distress)	E5,E7	Protection of vulnerable individuals; creates bonding through need for others
	E7: CARE system	E5,E6	Caring for others, particularly offspring
	E8: PLAY system	E6,E7	Bonding with conspecifics, development of basic adaptive, and social skills, creativity
Group function: regulating conflict	E9: POWER/dominance system (rank, status, submission)	E3,E4,E5	Limiting aggression in social groups: allocating resources, esp. sexual ones

*E1 is a generalized system providing incentive for the others and this dependence is noted only once. The systems are renumbered from Ellis and Toronchuk (2005), in line with our present scheme. The new numbering system will be retained in this paper*.

### Basic functioning

Panksepp has proposed that the SEEKING System is the primary task-oriented pathway by which affective goals are met (Panksepp, 1998). This generalized system activated by primary biological needs and characterized by homeostatic detection mechanisms, can also function in a non-specific manner. Other primary emotional systems (in our proposal labeled E3–E9), each characterized by a genre of intention and intensity of desire, feed information to the SEEKING system, as do secondary emotions thereby affecting overall motivational state^2^. Through inclusion of conscious volitional goals in E1, intentions and resulting purposive action gain emotive power: “I want that job,” “I need that house,” and so on, have affective components. This ultimately influences the way that ethical choices and values (the basis on which we choose acceptable actions) become effective in guiding action, as they too have underlying affective components.

Evidence now suggests (e.g., Berridge and Kringelbach, 2008; Berridge et al., 2009) that reward functions are parsed into two components – a motivational, appetitive system (corresponding to Panksepp’s SEEKING/expectancy system), and a distinct hedonic appraisal or consummatory system. This “wanting” vs “liking” distinction is supported by the fact that addiction involves craving but not necessarily satisfaction (Robinson and Berridge, 2003). The relevant neural pathways normally function together, but can be behaviorally dissociated (e.g., Knutson et al., 2001; Cannon and Bseikri, 2004), even in fish (Spector, 2000) suggesting the division is of ancient origins.

The earliest protovertebrate ancestors would of necessity had consummatory appraisal responses even before appetitive seeking behaviors evolved, hence “liking” might be considered the primordial reward system. In mammals the lowest level for hedonic appraisal must reside in the brainstem as decerebrate rats and anencephalic infants also show hedonic responses (Steiner et al., 2001). Although taste, which relies on medullary nuclei, was probably the earliest effective stimulus, the ventral pallidum, nucleus accumbens, and several other forebrain structures evolved to process and respond to pleasurable stimuli in many modalities. Thus in our model the multi-modal nature of hedonic appraisal parallels that of the general purpose SEEKING system described by Panksepp (see, e.g., Kelley and Berridge, 2002; Burgdorf and Panksepp, 2006).

The mesolimbic dopamine system, extending from midbrain ventral tegmental area (VTA), lateral hypothalamus, nucleus accumbens shell to orbitofrontal cortex, was traditionally implicated in the neural basis of reward; however it is now recognized that both “wanting” and “liking” mechanisms also utilize endogenous opioids (Levine and Billington, 2004; Peciña, 2008) and the ventral pallidum is a necessary component of the pathway (Smith et al., 2009). These systems play a fundamental role in learning, possibly by associating arousal with specific activities, thereby attaching a positive affective value to them and then acting as a stimulus for repetition of these activities (see, e.g., Wise, 2004; Rolls, 2005).

Following Panksepp’s (1998) suggestion that the SEEKING system evolved to provide a common currency of reward, we suggest that facilitated by associated hedonic appraisals there exists a primitive system shaping general coordination of affective responses. The dopamine system is activated not only by food, drugs, sex, electrical stimulation, and monetary reward, but also by aversive stimuli with response segregation according to positive or negative valence occurring in separate regions of the nucleus accumbens and pallidum (Kelley and Berridge, 2002; Smith et al., 2009). The ventral pallidum also responds to a wide variety of food, sexual, and affiliative cues (e.g., Rauch et al., 1999; Dillon et al., 2009; reviewed in Smith et al., 2009).

Psychological illnesses that Panksepp associates with malfunctioning of the SEEKING system include addictions and cravings, eating disorders, and possibly schizophrenia (see Panksepp, 1998; Panksepp and Harro, 2004). Panksepp (2002) considers obsessive compulsive behaviors to be malfunctions of this system, however, we suggest below that washing symptoms may arise instead from malfunctions of a primordial DISGUST system, while symptoms of checking and hoarding may arise from circuitry originating phylogenetically in a POWER/dominance system.

### Basic survival

In previous papers (Toronchuk and Ellis, 2007a,b) we described a primitive emotional system which, analogous to SEEKING, can be activated by various sensory modalities and ideational components, but with opposite functions to SEEKING. We designate this as the DISGUST system rather than AVOIDANCE or AVERSION because of the previous inclusion of disgust in various lists of basic emotions from Darwin onward. Together SEEKING and DISGUST would have been the primal opposing operating systems for ancestral organisms. In our model DISGUST evolved from primitive chemosensory mechanisms adapted to both avoid pathogens and their toxins and eject them from the gut if necessary. We propose this occurred in conjunction with the development of interaction between the immune and nervous systems (c.f. discussion in Ellis and Toronchuk, 2005; Rubio-Godoy et al., 2007) because non-specific (innate) immune cells are found in almost all multicelled organisms. Evolutionary adaptation would then lead to learned avoidance of toxic or infected material *before* ingestion, as opposed to vomiting afterward, ultimately giving rise to the subjective human experience of disgust as an anticipatory mechanism for avoidance.

The DISGUST system meets the criteria we enumerate above for primary emotional operating systems (Toronchuk and Ellis, 2007a,b, but see Panksepp, 2007). This system is intended as a parallel mechanism to appetitive SEEKING. Much broader than the sensory affect *distaste*, disgust is elicited by olfactory, gustatory, auditory, tactile, or visual cues (see Curtis and Biran, 2001; Curtis et al., 2004). Therefore it is not restricted to avoiding bad taste, but hinges on avoidance of contamination (Rozin and Fallon, 1987; Haidt et al., 1997; Rozin et al., 2009). Our proposal is that nutritional-, sexual-, and socially-related stimuli plus ideational components are all able to activate either the SEEKING or DISGUST systems in analogous ways.

We suggest that the disgust response did not arise merely as a reaction to bad taste, but due to association with increased likelihood of illness as proposed above (Curtis et al., 2004). Serotonin release is essential for development of disgust reactions and its use by the gut, vagus nerve, brainstem disgust mechanisms, and immune signaling suggests a defensive continuum of immune and disgust systems linked by serotonin (Rubio-Godoy et al., 2007). Negative hedonic value is not necessary for disgust as shown by conditioned taste aversions (CTA) elicited to sweet tastes paired with illness (e.g., Garcia et al., 1974; Parker et al., 2008). Conditioned immune responses may form from single pairings of novel taste with antigens (Pacheco-Lopez et al., 2004) further suggesting to us an evolutionary role for the immune system in disgust. Formation of a CTA requires activation of the insula (Ferreira et al., 2006) a structure which compares incoming and stored tastes (Koh and Bernstein, 2005). Rats with insular lesions fail to learn anticipatory discrimination although they remain capable of hedonic responses (Kesner and Gilbert, 2007).

Human anterior insula (AI), containing the gustatory cortex, also plays a role in self-awareness (reviewed in Toronchuk and Ellis, 2007a). It is activated during experience, observation, and imagination of disgust (Jabbi et al., 2008). Insular responses to aversive tastes vary according to expectations (Nitschke et al., 2006) and disgust sensitivity (Calder et al., 2007) as predicted for a primary emotional system. Although many behaviors integrating higher cognitive processing with “gut level” feelings also activate it (see Saper, 2002), AI does not provide a simple module for disgust but functions together with other areas including the basal ganglia to instantiate a variety of interoceptive bodily and conscious feeling states (Calder et al., 2001). The adjacent orbitofrontal cortex also plays a major role in taste, smell, and visceral responses and in assigning reward values to stimuli (Rolls, 2005; Rolls and Grabenhorst, 2008). Recent brain imaging research consistent with our proposal for separate DISGUST and SEEKING systems, suggests two regions of dorsolateral prefrontal cortex with different patterns of connectivity are differentially involved in approach and avoidance as parts of a network including orbitofrontal cortex, cingulate, amygdala, and basal ganglia (Spielberg et al., 2012).

Increased cortical integration of body states occurs in primates because the insula receives direct thalamocortical taste and visceral input which in rodents arrives indirectly via the amygdala (Craig, 2005). Emotional contagion or “resonance” is a further adaptation promoting disgust activation when observing socially relevant disgust responses in conspecifics (Wicker et al., 2003; von dem Hagen et al., 2009). The insula may also play a role in social evaluation cognition as a viscero-motor center which simulates the activity of others in a manner similar to “mirror” neurons previously described in monkeys (Gallese et al., 2004; Keysers and Gazzola, 2007). Cognitive processing allows primitive DISGUST impulses to be melded with social learning to produce secondary emotions incorporating social status and morality. Activation patterns of facial muscles in response to unpleasant tastes, contaminated objects, and unfair treatment are consistent with the suggestion of shared neural evaluative mechanisms for distaste, olfactory disgust and moral disgust (Chapman et al., 2009). The role of the insula in awareness of disgust in self and others, possibly facilitated by von Economo neurons in AI and adjacent orbitofrontal area, may be a preadaptation setting in motion the development of theory of mind and moral reasoning. We are thus suggesting that the primitive emotive circuit which originally functioned to defend the self by regulating consummatory behaviors contributes to an emotional system which facilitates some of the most highly developed human capacities of social evaluation (Toronchuk and Ellis, 2007a,b).

Disgust sensitivity is inversely correlated with sensation seeking (Haidt et al., 1994) further suggesting that DISGUST and SEEKING represent opposing operating systems which might then be reflected in contrasting behavioral traits and psychiatric disorders. Genetic influence in disgust sensitivity (Olatunji and Broman-Fulks, 2007; Kang et al., 2010) is consistent with the finding that even presymptomatic genetic carriers of Huntington’s disease show selective deficits in recognition of disgust (Hennenlotter et al., 2004) and insular size in presymptomatic patients is correlated with disgust recognition (Kipps et al., 2007). In contrast increased activation of the insula in OCD patients is associated with contamination, specifically washing-related symptoms (Stein et al., 2001; Shapira et al., 2003). Phillips and Mataix-Cols (2004) find that patterns of brain activation in OCD patients in response to disgust or anxiety-producing objects vary according to the patient’s major symptom type. Brain activation to disgust stimuli occurs independently of anxiety again supporting the view that OCD typified by contamination/washing symptoms represents an underlying malfunction of the DISGUST system (Husted et al., 2006; Lawrence et al., 2006).

In contrast to internal threat, protection from external sources was phylogenetically provided by the RAGE E3 and FEAR Systems E4 described in detail by Panksepp (1998). Previous experience and assessment of the present circumstances determines the final behavioral outcome of these systems in higher organisms. As with DISGUST certain stimuli are predisposed to easily activate FEAR (Öhman and Mineka, 2001) and as with DISGUST the same structures activated during the production of fear and anger are also activated in humans during recognition of fear and anger in others (Calder et al., 2001; see discussion in Goldman and Sripada, 2005). Panksepp suggests that certain psychological disorders such as aggression, psychopathic tendencies, and related personality disorders might be correlated with the malfunctioning of the RAGE system; while anxiety disorders, phobias, and PTSD variants might be associated with malfunctioning in the FEAR system (Panksepp, 2002).

### Reproduction

Sexual reproduction, an obvious necessity for evolutionary selection, is the outcome of the LUST system E5 which also infers “wanting” and “liking” components. As with the SEEKING system the appetitive and consummatory aspects can function independently with corresponding behavioral dissociation (Pfaus, 1996; Kippin et al., 2004). The transition from reptiles to mammals brought about modifications in this ancient system to produce the attachment necessary for lactation (Panksepp, 1998). Thus the circuitry for LUST is biochemically related to that of both PANIC/attachment (or separation) and CARE. Due to different mating strategies, the complex relationship between LUST, PANIC, and CARE likely differs in males and females giving rise to different attachment styles in adult humans (see Taylor, 2006; Del Giudice, 2009).

All the social emotional systems are mediated by hormones, synaptic signaling, and other biochemical signals as employed by other elements of the underlying primordial value systems. Social emotions can be therefore causally effective in terms of contributing to neural selection because these signals also affect brain plasticity. Oxytocin and vasopressin in particular function as neuromodulators in the value systems associated with both adult social bonds and parental behavior (reviewed in Carter, 1998, 2003). For example during mating vasopressin is released in the ventral pallidum and nucleus accumbens (areas associated with SEEKING) of male prairie voles; its blockade prevents pair bond formation (Lim and Young, 2004). In addition, vasopressin plays a role in sexual competition (see e.g., Sewards and Sewards, 2003a).

In parallel with the SEEKING system, dopamine is associated more with the appetitive phase of LUST and endogenous opioids more with the consummatory phase. Dopamine secretion increases with sexual arousal, but at consummation dopamine decreases while secretion of oxytocin and opioids increase (Pfaus, 1996; Van Ree et al., 2000). The neural circuitry of this system includes among other areas prefrontal, insular, and anterior cingulate cortex along with amygdalar, hypothalamic, and other subcortical areas (Stoléru et al., 2012). These cortical areas are highly interconnected with the amygdala and are also prominent in social bonding. The malfunctioning of this system might be reflected in fetishes and sexual addictions (Panksepp, 2002), and disorders of desire and orgasm resulting from either over-activation or under-activation of the subcomponents.

### Primary emotional systems of social bonding

CARE, PANIC, PLAY are also important emotions of social behavior discussed in detail by Panksepp (1998, 2002) Here we discuss primarily their crucial roles in sculpting mammalian social and cognitive development. According to MacLean (1990, p. 247) the differentiation of mammals from reptiles involved (1) lactation and associated maternal care, (2) vocal communication to maintain mother-infant contact, and (3) playful behavior facilitating social learning. Because lactation and maternal care are essential for mammalian survival, significant selection pressure would act on the neural mechanisms controlling these behaviors. In mammals social bonding is initially effected primarily by the PANIC/attachment (E6) system in the young, which triggers emotional panic during separation, but signals contentment during closeness. This necessitated the tandem development of a complementary CARE (E7) system, through which parents respond to the young. Panksepp’s (1998) PANIC system basically concerns separation distress; however, we suggest that distress is only one mode because the system also operates in a positive fashion when needs are satisfied. We propose that subjective feelings in the human infant include both panic/distress during separation, and also contentment/comfort during contact (E6); and in the care-giver, tenderness/affection, carrying over to reciprocal distress when the infant is perceived as in distress (E7).

The biological origins of human sadness may be rooted in an extended system used earlier in phylogeny for pain perception but which now includes the cingulate gyrus that mediates separation distress in infant mammals (Panksepp, 1998, 2003b). This origin is supported by the role of endogenous opioids in both pain reduction and positive feelings brought on by contact. Similar to the case of distaste and DISGUST, an early sensory affect (pain) perhaps gave rise to a basic emotional system (PANIC/attachment). Relational loss is thus perceived in humans as similar to pain and panic. Both adult and infant bonding are mediated by oxytocin (Carter, 1998, 2003; Insell and Young, 2001; Lim and Young, 2004; Taylor, 2006; Bos et al., 2012) perhaps also due to phylogenetic origin. Estrogen further enhances the effects of oxytocin, which may provide a basis for gender differences in human attachment styles (see Taylor, 2006; Del Giudice, 2009).

The long period of helplessness in hominid infants combined with a need for training in foraging and social behaviors likely put considerable selection pressure on the neural circuitry for emotional attachment between mother and infant. Ape mothers’ use of gestures and facial expressions plays a key role in communication with infants, who correspondingly develop an intense interest in the mother’s face Falk, 2004). Chimp mothers teach which foods are edible, and perhaps even tool use (Goodall, 1986; Falk, 2004). Chimp infants communicate various types of distress through specific vocalizations. Hominid evolution therefore involved tandem evolution of emotional circuitry in adults, in order to provide emotional nurturance and instruction, and parallel circuitry in the young to seek and respond to caregivers. This entailed increasing use of gesture, facial expression, tactile, and vocal communication. According to this scenario, the lengthening period of dependency in early humans due to increased brain size and subsequent immaturity at birth selected for even more skilled caretaking and teaching ability on the part of adults. Although the effects of early emotion on individual adult emotional and social behavior has been well-studied, the evolutionary role of infant emotion as a selection factor in cognitive development has been less well researched. According to the theory we suggest, mother-infant communication provided emotional motivation for the development of language. Learning in infants is critically enabled by reciprocal interaction with the care-giver (e.g., Schore, 1994). The ability for shared attention becomes necessary for the development of a theory of other minds and language use. Thus social emotions provide the valenced state necessary for infant learning, initially developed to predict and respond to actions and emotions of the care-giver. This provides a major example of how cognition is molded in part by emotion, and emotion in turn comes to be regulated by cognition.

Tactile stimulation in infancy mediates upregulation of glucocorticoid receptors in the hippocampus through DNA methylation, an effect which in rodents persists throughout life (Kaufman and Meaney, 2007). Upregulation decreases reactivity of the hypothalamic-pituitary-adrenal (HPA) axis via negative feedback. Early tactile stimulation of female rodents regulates expression of estrogen receptors in the medial preoptic area, resulting in adulthood in increased oxytocin receptor binding with subsequent increased licking, grooming, and nursing. Nurturant maternal care also enhances learning by enhancing NMDA receptor activity in the hippocampus. These mechanisms may all be conserved from rodents to humans (Kaufman and Meaney, 2007). Consistent with this, human adults who experienced childhood adversity both rate reward cues less positively and show less response in the reward-evaluating pallidum (Dillon et al., 2009). Together these effects illustrate the long-term influence of the PANIC and CARE circuitry on emotional and cognitive behaviors.

It seems worth investigating whether gender differences in stress responses (e.g., Dalla et al., 2008) might be related to demethylation of estrogen receptors in early life as described by Kauffman and Meaney (or other nurturant behaviors), thus affect attachment style. Specifically human males show more avoidant attachment, and females more ambivalent attachment from middle childhood through adulthood (Del Giudice, 2009). Fight and flight behavior is more typical of males under stress, while oxytocin facilitates tend and befriend behavior in females (Taylor, 2006).

Separation of infant and caretaker can lead to physical and emotional stunting as described by Rene Spitz in the 1940s, a concept that was later expanded by Harlow while the long term impact of maternal care on children was confirmed by Bowlby (Van der Horst and van der Veer, 2008). Panksepp predicts malfunctioning in these social systems, combined with other environmental factors, are associated with panic attacks, pathological grief, some types of depression, agoraphobia, social phobias, dependency, attachment disorders, and perhaps also be a contributing factor to autism (Panksepp, 2002). Nemeroff et al. (2006) seem to suggest an association with post-traumatic stress disorder. Thus, the PANIC and CARE systems facilitated evolution of human social bonding, altruistic behavior, and possibly influenced language evolution, while disruption is predicted to lead to numerous psychological disorders (see Toronchuk and Ellis, 2009).

### Learning and development

The interaction between emotion and cognition becomes especially apparent in the life-long processes involved in learning. Although learning is enabled by the primordial SEEKING system (see Berridge et al., 2009) it is ontologically dependent in mammals on the circuitry for PLAY E8. The importance of mammalian play as preparation for adult food procurement and social roles, suggests that play should be considered a basic emotional program necessary for normal human cognitive development. It was facilitated evolutionarily by cortical enlargement and the prolonged interaction necessitated by lactation (MacLean, 1990). Developmentally play is also influenced by the interaction between CARE and PANIC. Play involves learning social roles and behaviors (e.g., Bekoff and Byers, 1998; Brosnan, 2006; Keltner et al., 2006) and allows assessment of social commitments and dominance.

Extensive mother-infant play in chimps and especially bonobos is characterized by facial gestures, vocalizations, and laughter (reviewed in Falk, 2004). It is possible that optimal cortical plasticity depends on the rewarding effects provided by these interactions. Allowing juvenile rats 30 min of “rough and tumble play” results in increased BDNF transcription in the amygdala and dorsolateral frontal cortex (Gordon et al., 2003). Although much anecdotal information suggests an important role in human learning, the underlying neurophysiological mechanisms are not yet defined.

Panksepp (1998) includes “rough and tumble play” in the list of primary emotions, but we speculate that in humans the neural activation has been extended to facilitate representational and intellectually imaginative play. Evidence exists that both captive and wild apes may engage in representational play in which one object comes to stand for another (Lyn et al., 2006). We conjecture that the phylogenetic transition from “rough and tumble play” to representational play facilitated the evolution of language through development of a mechanism allowing symbolic representation. A theory of other minds, another factor in imaginative play, is likely necessary for language evolution. Play thus remains particularly important in the ontogenetic processes of language development (see Bruner, 1983; Paley, 2004; Zigler et al., 2004).

Many aspects of human culture such as the performing arts, ceremonial, and celebratory behavior, and even creativity in science ultimately depend on extensions of these mechanisms (see Frost et al., 2001). Humor with embedded dominance paradigms in which we “make fun” of others could be another expression of rough and tumble activity. Social aspects such as turn taking and feinting may also contribute to development of altruistic behavior. A detailed assessment of the PLAY system, and the associated role of opioids, is given by Panksepp (1998, 2002). He suggests that attention-deficit hyperactivity disorder, mania, and perhaps impulse control disorders may be associated with its malfunctioning.

### Group function: Regulating conflict and competition

A main point of the present paper is to propose that a primordial POWER/dominance System, concerned with territoriality, dominance, and subordination should be added to the list of basic emotional operating circuits. Several recent studies discussed below are consistent with the presence in humans of a neural system involved in the determination of status (reviewed in Beasley et al., 2012). The widespread existence of dominance displays among vertebrates suggests ancient evolutionary origins. Group living may enhance individual survival via cooperative food procurement, protection, and learning, but also entails competition for resources. Selection pressure therefore likely favored the development of mechanisms which communicated the dominant status of some individuals, while allowing survival of those subordinates who participate in cooperative group activities providing them with a chance for future reproduction. Panksepp (1998, 2002, 2007) describes social dominance as arising from interactions between the neural circuitry underlying childhood PLAY and that underlying adult FEAR and RAGE. Although ontogenetic development of dominance in individuals is often enabled by play, we point out that dominance is phylogenetically much more ancient than play. According to MacLean (1990) play was first important among mammals.

Allocation of rank leads to agonic behavior which regulates competition while minimizing tension (e.g., De Waal, 1996, pp. 89–125; Stevens and Price, 2002, pp. 49–52). Human territorial and sexual competition has been extended to include material resources, social control, status symbols, and intellectual turf. Personal identity, therefore, comes to be closely influenced by “territorial” tendencies^3^. Based on data from psychiatry and ethology, we propose that the ancient system of dominance and submissive subroutines is the phylogenetic precursor to competition for status, the need to excel, and obtain social approval. With a few modifications it is similar to the “power dominance” drive that Sewards and Sewards (2003b) propose as foundational to Nietzsche’s will to power and Winter’s (1973) implicit power motive.

Price et al. (2007) point out that elevated mood facilitates a rise in rank which enables coping with leadership, while depressed mood allows lower ranking individuals to communicate acceptance of their status and forgo reward. The desire for higher rank is associated with feelings of pride/high self-esteem during success and shame/low self-esteem or depression during defeat^4^. They locate the instinctual aspects of depression as largely a function of the striatal complex of MacLean’s reptilian brain. This area was responsible for ancient instinctual behavior patterns used in reptilian display, while limbic structures were only recruited after the evolution of mammals (MacLean, 1990).

The striatal complex, particularly the basal ganglia, plays a major role in vertebrate dominance behaviors. Its activation occurs during social/territorial displays among male lizards along with release of serotonin and dopamine in different patterns correlated with dominance and subordination (MacLean, 1990; Baxter, 2001). The localization of serotonin and dopamine is similar in lizard and primate basal ganglia inferring similar mechanisms (Baxter, 2003). Lesions in globus pallidus of the mammalian basal ganglia disrupt dominance and courtship displays given by male monkeys (MacLean, 1990; Newman, 2003) while non-sexual competitive arousal activates the human ventral pallidum associated with reward (Rauch et al., 1999). The ventral striatum has also been shown to be active in humans during value assessments of socioeconomic status (Ly et al., 2011). The basal ganglia, as Price suggests, may mediate those components of depression which act as appeasement displays after loss of agonistic encounters. The role of the ventral pallidum in reward assessment (Smith et al., 2009) may thus be relevant to this type of depression.

In addition to the striatal system, the ventromedial prefrontal, anterior cingulate cortices (ACC), and amygdala are also involved in the POWER system of humans (Beasley et al., 2012). Various prefrontal areas contribute to perception of status (Karafin et al., 2004; Zink et al., 2008; Marsh et al., 2009) and also in OCD (Baxter, 2003; Blackford and Pine, 2012). The prefrontal and nearby anterior cingulate areas have multiple diverse connections to the amygdala which allow for both top-down and bottom-up regulation (Ray and Zald, 2012). The amygdala thus provides non-conscious regulation of these cortical areas. ACC is activated during competitive arousal (Rauch et al., 1999); and low activity here is implicated in the pathogenesis of depression with different subregions of ACC playing differential roles (see Davidson et al., 2002). Sewards and Sewards (2002, 2003b) describe an area of ACC (referred to as BA 32) as participating in the “power dominance” drive and equate this area with the infralimbic area identified in hamsters as responsible for dominance (Kollack-Walker et al., 1999). Stewards and Stewards propose the major contribution of this area is to the learned, voluntary components of the power dominance drive rather than the involuntary components which are mediated elsewhere. Volumetric analysis of an area including BA32 reveals a negative correlation with self-reports of perceived social status, without a corresponding change in amygdala (Gianaros et al., 2007). In Price’s model, following MacLean, these involuntary components would be controlled by the basal ganglia.

Activation of submissive circuitry may lead to depression (see Price, 1967; Gilbert, 1992; Stevens and Price, 2002; Price et al., 2004, 2007), which is a possible human counterpart of social defeat (e.g., Weisfeld and Wendorf, 2000; Sloman et al., 2003; Kroes et al., 2007). Low status is known to be a major risk factor for depressive behavior (e.g., Gilbert and Allan, 1998; Panksepp et al., 2002) and adverse health effects in humans and other primates (Sapolsky, 2005), and according to MacLean even in reptiles.

Biochemical activation of this circuitry occurs in part by serotonin, which plays a general background role in numerous emotional systems, and may increase dominance by decreasing impulsive, species-typical behaviors. Low serotonin levels are reported in numerous psychiatric disorders including depression, suicide, anxiety, aggression, addiction, and OCD. Dominance behaviors and serotonin levels influence one another in both vertebrates and invertebrates (e.g., crayfish, Panksepp and Huber, 2002). An example from *Anolis* lizards is that manipulation of dominance relationships leads to changes in serotonin levels in several brain areas including basal ganglia (Korzan and Summers, 2004). In hamsters dominance status is also reported to affect serotonin transporter expression (Morrison et al., 2011). Serotonin levels correlate with rank order in vervet monkeys; social standing among individual monkeys has been manipulated by altering its level (Raleigh et al., 1991). Increasing serotonin by means of oral tryptophan increases dominance behavior in human males (Moskowitz et al., 2003) while decreasing serotonin reuptake reduces submissive behaviors.

Several hormones and neuropeptides also play direct roles even in human dominance behaviors (reviewed by Bos et al., 2012). Testosterone increases dominance behaviors in humans (Archer, 2006) and macaques (Rilling et al., 2004), and it is suggested the effects in both sexes probably correlate more with status behaviors than direct aggression (Eisenegger et al., 2010). Although testosterone may increase competitiveness and dominance, high testosterone accompanied by low serotonin may lead to impulsivity and therefore pathological aggression (Birger et al., 2003). Social defeat in rodents (Panksepp et al., 2007) results in widespread decreases in cholecystokinin (CCK) while play behavior produces increased levels of CCK (Burgdorf and Panksepp, 2006). In these rats social defeat also increased levels of corticotrophin releasing hormone (CRH), a change otherwise associated with depression (see Brown et al., 2004). Another biochemical similarity between animal social defeat and human depression is the finding that interleukin 18, over-expressed in depressed humans, is also increased following social defeat in rats (Panksepp et al., 2007).

Vasopressin, and its non-mammalian homolog vasotocin, facilitates aggressive and dominance related behaviors in many species and is itself modulated by testosterone and serotonin (reviewed by Bos et al., 2012). In human males vasopressin administration modulates activity and effective connectivity in the subgenual cingulate cortex during processing of facial emotions by abolishing fear-related decreases in cingulate activity (Zink et al., 2010). Zink et al. also suggest this cingulate area might be involved in a negative feedback loop with the amygdala. Increased vasopressin is further linked to human OCD (Altemus et al., 1992); and may play a role in some forms of stress-related depression (Landgraf, 2006). Sewards and Sewards (2003a,b) proposed that vasopressin is the primary generator of the “power dominance” drive. Given experimentally to men and women, however, it has a differential effect, producing increased agonistic behavior in men and increased affiliative behavior in women (Thompson et al., 2006), again suggesting gender differences in the normal development of aggression and nurturant-related neural circuitry. Social subjugation of hamsters decreases levels of vasopressin in the anterior hypothalamus (Delville et al., 1998). In contrast microinjection into this area triggers stereotypical behaviors (grooming and scent marking) thought to model human OCD but these behaviors are suppressed by drugs used in its treatment (Ferris et al., 2001).

Taken together the involvement of the basal ganglia, vasopressin, and serotonin in social dominance and OCD may be consistent with the view of Stevens and Price (2002) that some forms of OCD can be a disorder of POWER/dominance. MacLean originally proposed that some OCD might be the result of inappropriate release of territorial and defensive motor program fragments underlying checking and perhaps hoarding, but not washing-type symptoms of OCD. A second hypothesis regarding POWER is that at times depression might result from triggering the involuntary subordination response of animals losing competitive encounters, i.e., some forms of depression may also represent disorders of rank (e.g., Gilbert, 1992; Gilbert and Allan, 1998; Sloman, 2000). While it has been further suggested that the involuntary subordination response may have phylogenetic origins in separation distress because both are characterized by negative mood, loss of self-confidence, and result in decreased activity (Sloman et al., 2003) however, it should be pointed out that the ancient dominance and subordination system is also present in reptiles with no known attachment systems.

Weisfeld and Wendorf (2000), building on Beck’s differentiation of sociotropy and autonomy, propose that depression assumes two forms. Sociotropy, they suggest, is a personality characteristic related to attachment and the need to please others, while they describe autonomy as related to independence and attainment of goals. Depression which results from loss of status and often involving pride, guilt, and/or shame might therefore be differentiated from depression associated with grief or loneliness. These two characteristics are expressed in different symptoms (Robins et al., 1989) with autonomous traits more likely to respond better to antidepressant medication than sociotropic traits (Peselow et al., 1992). Also in the non-clinical population low moods are associated with different behaviors depending on whether such moods are precipitated by social losses or failure to reach a goal (Keller and Nesse, 2005).

Consistent with the notion of two forms of depression discussed above, the physiology and the developmental course of depression might differ according to whether it is precipitated by separation distress or by loss of status. In line with this notion, oxytocin reduces separation distress, but not low rank; separation anxiety involves parts of cingulate cortex, while in humans, dominance perception, pride, and shame might more preferentially involve components of orbitofrontal/prefrontal cortex (Panksepp, 1998; Weisfeld and Wendorf, 2000; Karafin et al., 2004; Marsh et al., 2009). Another study suggesting two forms of depression differentiated by social components, reported left frontal activation in depressives scoring high in reassurance-seeking, but relative right frontal activation in those low in reassurance-seeking (Minnix et al., 2004. Although speculative and not necessary for our theory, differential laterality may relate to Gray’s (1987, 1990) BAS/BIS distinction or Buck’s (1999) selfish vs. social division. Weisfeld and Wendorf note the similarity between subordination displays and the behavior of individuals whose depression is associated with shame, guilt, and failure, e.g., averted gaze, slumped posture, and slowed responses.

In summary, we propose that in addition to the systems listed by Panksepp, there is a genetically determined emotional operating system which influences dominance and subordination with instinctual motor components based in the basal ganglia and emotional components based in limbic structures and modulated by cingulate and prefrontal areas. The development of neocortex allowed for integration with cognition leading to the emergence of more precise human secondary emotions such as guilt, shame and jealousy. We propose malfunctioning of this POWER/dominance system might take two forms: over-activation of subordination programs might result in depression; over-activation of dominance programs might be related to checking, ordering, and hoarding symptoms of OCD. In contrast OCD with washing symptoms more likely arises from malfunction in the DISGUST system as proposed above; while we suggest depression with sociotropic symptoms is mediated by the PANIC/attachment system.

## Conclusion

According to the proposals of Affective Neuronal Selection, the primordial emotional operating systems play a major role in determining the details of higher brain functions such as cognition and secondary emotions. We think it therefore useful to use the criteria C1–C7 above as assessment tools in analysis of the *necessary and sufficient* variables underlying later brain development (Ellis and Toronchuk, 2005). A summary of the operating systems we include is given in Table 3. On the basis of evolutionary we have added (a) the DISGUST system E2 and (b) the POWER/dominance system E9 to those included in our previous paper (based on Panksepp, 1998).

**Table 2:** **The proposed basic emotional systems together with their associated brain areas and key neuromodulators**.

Evolutionary needs met	Primary emotional system	Putative neurochemicals	Putative key components of neural networks
**INDIVIDUAL NEEDS**			
Basic functioning	E1: SEEKING system (hedonic appraisal, ”liking” component)	Endorphins (+), GABA (+, −) enkephalins, DA (?) endocannabinoids (+)	Nucleus accumbens, ventral pallidum, VTA, brainstem nuclei
	E1: SEEKING system (incentive motivation “wanting” component)	DA (+), glutamate, Ach, CCK (+, −), neurotensin, endorphins	Nucleus accumbens, ventral pallidum, lateral hypothalamus, and VTA to PAG
Basic survival	E2: DISGUST System (repulsion)	Serotonin (+), substance P (+)? Endocannabinoids (−)	Anterior insula, putamen, lower brainstem (area postrema, NTS)
	E3: RAGE System	Substance P (+), Ach (+), Glutamate (+), Vasopressin (+)	Medial amygdala, BNST, medial and perifornical hypothalamus, dorsal PAG
	E4: FEAR system	Glutamate (+), DBI, CRH (+), CCK (+),(α-MSH, NPY	Lateral and central amygdala, medial and anterior hypothalamus to dorsal PAG and pontine nuclei
**SOCIAL NEEDS**			
Reproduction	E5: LUST System Sexual desire	Steroids (+), Vasopressin (+), LHRH (+), DA (+)	Basal forebrain, amygdala, BNST, anterior cingulate, medial preoptic, and VMH to ventral PAG
	Sexual satisfaction	Opioids (+), Oxytocin (+)	Septum, medial preoptic (VMH in 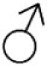 ?), VTA to PAG
Group cohesion: bonding and development	E6: NEED/ATTACHMENT (separation distress)	Opioids (−, +), oxytocin (−, +), prolactin (−/+), CRH	Anterior cingulate, BNST, POA, VTA, to PAG
	E7: CARE/nurturance	Oxytocin (+), prolactin (+), dopamine, opioids (±) glutamate (+)	Anterior cingulate, BNST, preoptic hypothalamus, to VTA and PAG
	E8: PLAY System	Opioids (+, −), DA (+) Ach (()	Dorso-medial diencephalon (thalamic nuclei) to ventral PAG
Group function: regulating conflict	E9: POWER/dominance (rank, status, submission)	Serotonin (±), DA (±) testosterone (±) vasopressin (±) CCK. CRH (±)	Medial prefrontal cortex, ventral pallidum, and other basal ganglia, hypothalamic nuclei to PAG

*The non-specific effects of serotonin and norepinephrine, are omitted, as are higher cortical areas. Based on Panksepp and Harro (2004), Watt (1999), Panksepp and Harro (2004), Watt (1999) and sources referenced throughout the text. Key: CCK, cholecystokinin; CRH, corticotrophin releasing hormone; DA, dopamine; DBI, diazepam binding inhibitor; LH-RH, leutenizing hormone releasing hormone; MSH, melanocyte stimulating hormone; NPY, neuropeptide Y; PAG, periaqueductal gray; BNST, bed nucleus of the stria terminalis; NTS, nucleus tractus solitarius; POA, preoptic area; VMH, ventromedial hypothalamus; VTA, ventral tegmental area*.

**Table 3:** **Satisfaction of criteria C1–C7 for Basic Emotional Systems by the proposed primary emotional systems E1–E9, as we understand them on the basis of data presently available**.

Primary emotional system	Criteria for basic system substantially satisfied? [criteria numbered as in section 3]						
	C1	C2	C3	C4	C5	C6	C7
E0/E1: Pleasure and SEEKING (satisfaction and incentive salience)	Yes	Yes	Yes	Partly	Yes	Yes	Yes
E2: DISGUST system (repulsion)	Yes	Yes	Partly	Partly	Yes	Yes	Yes
E3: RAGE system	Yes	Yes	Yes	Not yet	Yes	Yes	Yes
E4: FEAR System	Yes	Yes	Yes	Partly	Yes	Yes	Yes
E5: LUST system	Yes	Yes	Yes	Not yet	Yes	Yes	Yes
E6: PANIC/attachment (affiliation, separation distress)^‡^	Yes	Yes	Yes	Partly	Yes	Yes	Yes
E7: CARE system	Yes	Yes	Yes	Not yet	Yes	Yes	Yes
E8: PLAY system	Yes	Partly	Partly	Not yet	Yes	Yes	Yes
E9: POWER/dominance system (rank, status, submission)	Yes	Partly	Partly	Partly	Yes	Yes	Yes

*The criteria are C1, Concept (see Tables 1– 2); C2, Structure (neuroanatomy, see Table 2); C3, Function (neurotransmitters, see Table 2); C4, Development (genetics); C5, Evolutionary Origin (see Table 1); C6, Occurrence (homologs, see main text); C7, Outcome (psychiatric outcomes, see main text)*.

Since the time of Darwin (e.g., Tomkins, 1962; Ekman, 1972, 1992; Izard, 1992; Damasio, 2003; Plutchik, 2003) various lists of so called human “basic emotions” have been suggested. Our proposal is unique in seeking to characterize those phylogenetically influenced primordial systems which influence neural development and thereby influence later behavior and disorders. Virtually all previous lists of such basic human emotions include Happiness, Sadness, Fear, and Anger, and most include Disgust and/or Contempt. Some suggest Surprise and Interest, or less frequently Guilt or Shame. In our model Panksepp’s PANIC executive system (Panksepp, 2003b) underlies sadness by mediating attachment and pain of separation. We argue that it is useful when referring to operating systems rather than human affects to parse SEEKING into incentive salience and hedonic appraisal, two components which may be separately accessed by other emotions (Berridge and Robinson, 2003; Berridge et al., 2009). While the PLAY executive system is certainly associated with joy or happiness (Panksepp, 1998), we suggest happiness is more broadly based and corresponds to generalized hedonic appraisal based in “liking” and interest in the “wanting” subsystem.

In contrast, we do not consider Surprise to be a primordial system, for despite its inclusion in many lists of basic human emotions, it does not have the same nature as other affect programs (Griffiths, 1997, p. 241), is not necessarily valenced (Ortony and Turner, 1990; Ekman, 1992; Prinz, 2004, p. 163), and gives no specific action guidance for survival. However, like the startle reflex, it may serve to activate the SEEKING circuitry. We do not consider contempt, embarrassment, shame, and guilt as candidates for emotional organizing systems because firstly they are uniquely human; and secondly they rely largely on neocortical functions and so are more plausibly secondary emotions. Shame and guilt are likely secondary emotions emerging from cognitive interactions with the POWER/dominance circuitry (see Gilbert and Allan, 1998). Sabini and Silver (2005) argue for the inclusion of love and jealousy as basic emotions on the basis of their evolutionary past, however the terms CARE and POWER/dominance are more consistent with our concept of *emotional operating systems* reflecting developmental and evolutionary origins.

Although some have argued that the concept of basic emotions is not useful (e.g., Ortony and Turner, 1990), there are three qualified advantages from our perspective. First we see the operating systems underlying certain key emotions playing a key role in brain evolution and development (Affective Neural Selection), a proposal which may generate future research on evolutionary and developmental processes. Secondly, focusing attention on the functions of these primordial systems in the evolutionary past has led psychology to a greater awareness of the biological complexities of human emotions. This promotes research on new treatments and diagnostic techniques. For example the two subtypes of depression and OCD that we have described suggest not only different pharmaceutical treatments, but possible development of differentiating diagnostic techniques based on CHR or steroid levels, fMRI or genetic testing. Finally, along with the concept of Affective Neural Selection, this modified concept of primordial emotional systems may lead to new ideas on the promotion of healthy emotional development in infancy, and perhaps prenatal treatment of certain genetic variants.

Causal links are supported by the suggestions made throughout this paper for psychiatric disorders associated with each of the proposed primary emotional systems. Developing and validating those proposals will be an important future step. Table 3 shows our view on the current state of confirmation of the criteria C1–C7. Elucidation of genetic links will be important in validating the model, although this task is hampered by the polygenic nature of the common heritable mental disorders and the relative rarity of each of a very large number of specific mutations contributing to each disorder (Keller and Miller, 2006).

### Implications

The nature of the phylogenetic influence on emotional behavior has consequences in many areas of human life, e.g., economics, politics, and education (Ellis, 2008). Given the assumptions of Affective Neural Selection, we have a more nuanced version of motivational theory than simple conditioning theory provides. Each major survival and reproductive need, we propose, is related to one or more of the specific primary emotional systems either directly, or indirectly through secondary emotions; hence we can analyze psychological and psychiatric issues in terms of their relation to these primary operating systems in order to develop new forms of both therapy and prevention.

### Further steps

The proposals here represent a small step toward developing the psychological implications of Affective Neural Selection. Further key steps include

1. Validating the *list of primary phylogenetically influenced emotional operating system* (Tables 1 and 2) for correctness and completeness. Is each case for inclusion adequate (cf. Table 3)? The model can be supported or corrected by continuing research on the links between the prototype states, neuroplasticity molecules and genes (for example Kroes et al., 2006). Our model also predicts that activation of different primary systems (e.g., PANIC/attachment vs. POWER/subordination in depressive patients) during ongoing evaluative tasks and emotion regulation will be reflected in differential activity in subcortical brain structures.
2. Using the resulting list of system to determine and classify the *nature of secondary emotions* arising out of these primary systems via the processes of Affective Neural Selection. Further research should be undertaken to elucidate the influence of secondary emotions on cognition.
3. Further elucidation of the effects of phylogenetically and ontologically primitive emotions on the cognitive development of individuals. Our proposal is consistent with the notion that even small alterations in prenatal environment due to cytokines and transmitters may produce large changes in cognitive development. Further research is necessary along these lines.

These steps relate to the broader task of clarifying the issue of *psychological universals*. Human commonalities and differences develop in the context of societies that have universal functional needs and physical environments with commonalities based on universal underlying physical laws. In examining the function of the human mind, the emotional systems must be taken in conjunction on the one hand with the constellation of systems for perception, pattern recognition, and memory, and on the other the mechanisms of volition that balance rationality with the unconscious, emotion, and value systems. Understanding these interactions should lead to an enhanced understanding of the evolutionary and developmental basis of emotional disorders (Price, 1967; Panksepp, 2002; Stevens and Price, 2002) taking cognizance of neuroscience discoveries, current psychiatric knowledge, animal behavior, and neurology.

Finally, the question arises as to what difference there is between our proposal of Affective Neural Selection and the Hebbian processes of neural refinement on one hand and operant conditioning on the other. A summary of the differences is shown in Table 4. Note that Affective Neural Selection includes Skinnerian conditioning as a special case; but flexible and nuanced. It also works with Hebbian processes, but gives synaptic refinement a valenced or value-based direction that pure Hebbian processes lack. The overall key issue is the usefulness of a multi-dimensional affective assessment as proposed here, rather than a one-dimensional system as given by operant conditioning. The basic answer is that survival is enhanced in a complex environment that has been already sampled numerous times during evolutionary history, with the resultant lessons encapsulated in swift emotional reactions to various survival problems (see Table 1). These then shape development of ongoing brain functions during the entire life span. In brief: it enables organisms to benefit directly from the survival lessons experienced by evolutionary ancestors. In this sense it can be conceptualized as a precursor of cultural evolution. Detailed demonstration is now needed as to how this leads to enhanced survival prospects in early life before much learning has occurred.

**Table 4:** **Comparison of Hebbian and Skinnerian processes with Affective Neural Selection**.

Neural Processes	Activity dependent neural refinement	Response to overall affective state	Further genetically-based value system dimensions	Overall valence/value system
Hebbian processes	Yes	No	No	None
Skinnerian conditioning	Yes	Yes	No	One-dimensional
Affective neural selection	Yes	Yes	Yes	Nine-dimensional (see Table 1)

*The Overall Affective State (Column 3) provides a 1-dimensional assessment of the organism’s state (positive or negative; pain or pleasure). The genetically-based Value System Dimensions (Column 4) in the case of AND incorporate nuanced survival information selected during evolutionary history and transmitted genetically. Thus these encode specific inbuilt behavior tendencies that are appropriate in different circumstances and are available without prior learning. The Overall Valence/Value System (Column 5) operating in the case of Affective Neural Selection incorporates emotional/affective evaluations, and so relates to the importance of emotional systems in behavior, survival, and hence in evolution. These effects are not present in the cases of either simple Hebbian processes or Skinnerian conditioning*.

Overall, our proposal is supported by a growing number of studies emphasizing the role of emotions in the ontogeny and phylogeny of human cognition. Greenspan and Shanker, 2004, p. 1) have thus aptly encapsulated our approach: “*We have found that the capacity to create symbols and to think stems from what was often thought of by philosophers as the ‘enemy’ of reason and logic: our passions and emotions … we will show how emotions actually give birth to our very ability to create symbols and to think*.”

## Conflict of Interest Statement

The authors declare that the research was conducted in the absence of any commercial or financial relationships that could be construed as a potential conflict of interest.

## Footnotes

^1^The numbering system E1 to E7 is introduced in Ellis and Toronchuk (2005) although we have changed the ordering in this paper. Panksepp uses capitalization to denote emotional organizing systems rather than emotions *per se* and we retain this designation.

^2^While it is possible for each goal to have separate seeking and pleasure systems, resulting in a combinatorial increase of complexity, it is more economical to have many systems utilizing the same superordinate seeking and pleasure systems. The dopamine and opiate pathways are both generalized and responsive to many stimuli (see Berridge, 2004; Smith et al., 2009).

^3^We thank Ian MacCallum for pointing this out.

^4^Guilt, however, relates to failure to live up to expectations of self or others; thus it is a secondary emotion-related to ethical behavior rather than to ranking (see discussion below).
